# The effects of oviposition-site deprivation on *Anopheles gambiae* reproduction

**DOI:** 10.1186/1756-3305-5-235

**Published:** 2012-10-16

**Authors:** Kathryne L Dieter, Diana L Huestis, Tovi Lehmann

**Affiliations:** 1 Laboratory of Malaria and Vector Research, National Institute of Allergy and Infectious Diseases, National Institutes of Health, 12735 Twinbrook Parkway, Room 2W-09-C, Rockville, MD, 20852, USA

**Keywords:** *Anopheles gambiae*, Dry season, Drought, Hatch rate, Malaria, Reproduction, Seasonality, Vectorial capacity, Oviposition, Fecundity

## Abstract

**Background:**

The African malaria mosquito, *Anopheles gambiae*, depends on availability of suitable surface water for oviposition. Short and long dry spells occur throughout the year in many parts of its range that limit its access to oviposition sites. Although not well understood, oviposition-site deprivation has been found to rapidly reduce egg batch size and hatch rate of several mosquito species. We conducted laboratory experiments to assess these effects of oviposition-site deprivation on *An. gambiae* and to evaluate the role of nutrition and sperm viability as mediators of these effects.

**Methods:**

*Anopheles gambiae* adults (1–2 d old) from the G3 laboratory colony were assigned to the following treatment groups: oviposition-deprived (fed once and then deprived of oviposition site for 7 or 14 d), multiple-fed control (fed regularly once a week and allowed to lay eggs without delay), and age matched blood-deprived control (fed once, three days before water for oviposition was provided). Egg batch size and hatch rate were measured. In the second experiment two additional treatment groups were included: oviposition-deprived females that received either a second (supplemental) blood meal or virgin males (supplemental mating) 4 days prior to receiving water for oviposition.

**Results:**

*An. gambiae* was highly sensitive to oviposition-site deprivation. Egg batch size dropped sharply to 0–3.5 egg/female within 14 days, due to reduced oviposition rate rather than a reduced number of eggs/batch. Egg hatch rate also fell dramatically to 0-2% within 7 days. The frequency of brown eggs that fail to tan was elevated. A supplemental blood meal, but not ‘supplemental insemination,’ recovered the oviposition rate of females subjected to oviposition-site deprivation. Similarly, a supplemental blood meal, but not ‘supplemental insemination,’ partly recovered hatch rate, but this increase was marginally significant (P < 0.069).

**Conclusions:**

Even a short dry spell resulting in oviposition-site deprivation for several days may result in a dramatic decline of *An. gambiae* populations via reduced fecundity and fertility. However, females taking supplemental blood meals regain at least some reproductive success. If mosquitoes subjected to oviposition-site deprivation increase the frequency of blood feeding, malaria transmission may even increase during a short dry spell. The relevance of oviposition-site deprivation as a cue to alter the physiology of *An. gambiae* during the long dry season is not evident from these results because no reduction in hatch rate was evident in wild M-form *An. gambiae* collected in the dry season in the Sahel by previous studies.

## Background

Like most mosquitoes, *Anopheles gambiae*, the African malaria mosquito, depends on availability of suitable surface water for oviposition. However, during the dry season and dry spells during the wet season, larval sites may not be available for days, weeks, or even months in different environments [1-6]. Under such conditions, mosquitoes that develop eggs cannot lay them, a state hereafter referred to as “oviposition-site deprivation” [7], which differs from ‘egg retention’, a term applied also to an inseminated female which does not lay (some) developed eggs despite having access to a suitable oviposition site. The effect of oviposition-site deprivation on the physiology, behavior, and future life history of mosquitoes are scantly known, despite their possible ecological and public health implications. For example, the effects of oviposition-site deprivation on reproductive capacity, feeding rate, and longevity may correspondingly affect population size and disease transmission intensity during and following the period of deprivation. Moreover, not finding suitable larval sites may serve as a signal used by mosquitoes to switch from the typical reproductive state to reproductive depression in the dry season as previously described [8-14].

Only a few studies have evaluated the effects of oviposition-site deprivation on life-history traits. Reduced egg-batch size and hatch rate following oviposition-site deprivation was observed in *Aedes aegypti*, *Ae. albopictus*, *Culex quinquefasciatus*, *An. maculatus*, and *An. pharoensis*[7,15-19]. In these studies, the duration of oviposition-site deprivation varied from a mere 2 days (*An. pharoensis*) to 10 weeks (*Ae. albopictus* and *Cu. quinquefasciatus*), however, statistical analysis was not always applied to the data. Both *An. pharoensis*[15] and *An. maculatus*[18] revealed increased sensitivity of anophelines to short-term oviposition-site deprivation (2–5 d) compared with culicine species (10–70 d), as measured by their egg batch size and hatch rate over shorter periods of deprivation.

Here we evaluate the effects of oviposition-site deprivation on the egg batch size and hatch rate of *An. gambiae* under laboratory conditions. We attempt to separate the effects of aging due to time and due to cumulative gonotrophic cycles from the oviposition-site deprivation itself and also evaluate the effect of sperm viability and the female nutritional state on the response to oviposition-site deprivation. Because this species experiences oviposition-site deprivation naturally, we hypothesized it would be less sensitive than other anopheline species previously examined. Our results suggest, however, that even short-term oviposition-site deprivation reduces egg batch size and hatch rate of *An. gambiae* and that nutrition is a key mediator of this effect.

## Methods

### Experimental design

**Figure 1:**
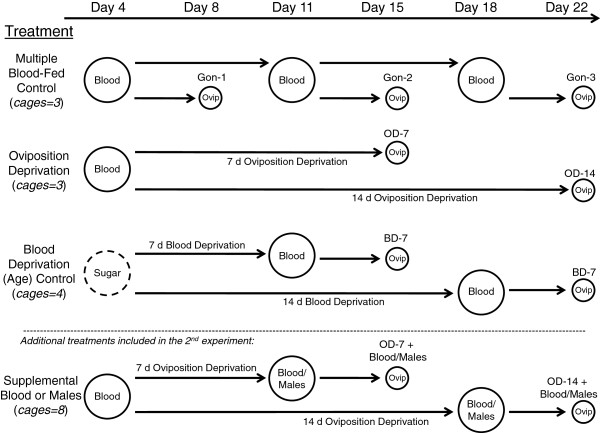
**Schematic showing the basic experimental design with the time line above and treatments listed on the right.** Large circles represent replicate cages (all set up on the same day with 100 females and 100 males each), with number of replicate cages per treatment in each experiment shown. Small circles represent individual oviposition tubes, where females were placed and provided water and filter-paper for oviposition (8 females per cage). In the design of the second experiment, two treatments, (shown below the dotted line) consisting of either a supplemental blood meal or supplemental males for insemination, were included in addition to those used in the first experiment (shown above the dotted line).

The G3 colony of *An. gambiae* was used throughout. Mosquitoes were reared under standard insectary conditions (27°C, 75% RH, L:D 12:12 hrs). Adults received fresh sugar solution daily. In each experiment, a total of 2,000 mosquitoes were divided, at 1–2 days old, into cages consisting of 100 females and 100 males to allow mating (Figure 1). Three cages were randomly assigned to the multiple-fed control, three cages for the oviposition-deprived group, and four cages for the blood-deprived group (two each for the once-fed on day 11 and once-fed on day 18 controls, Figure 1). When females were four days old (11 or 18 days for the once-fed controls), white leghorn chickens (*Gallus gallus*) were used to blood feed for twenty minutes; mosquitoes were provided with an additional twenty minutes if the feeding rate was <90%. Two-3-month old chickens were used to feed the mosquitoes in the cages, each one for a complete set of replicate cages (across all treatments). Unfed females were removed six hours after blood feeding.

On day seven, females to be assayed for oviposition (8–10 females/replicate cage of each treatment) were separated into individual 50 mL conical tubes lined with absorbent paper for egg laying and secured with netting on top to allow access to sugar pads. Males were also removed from all cages on day seven. Water for oviposition (10 mL of dechlorinated tap water) was added individually to the tubes four days after blood feeding (no oviposition-site deprivation), 11 days after blood feeding (short-term: 7-day oviposition-site deprivation), or 18 days after blood feeding (long term: 14-day oviposition-site deprivation). Control groups included females that were blood-fed regularly (once a week on days 4, 11, and 18) and those that were blood-fed only once (four days before water for oviposition was provided). Egg dishes were placed in the cages of the multiple-fed controls to allow the remaining females to lay eggs. Females that did not receive a blood meal on day 4 were considered blood-deprived (BD). These groups were fed once on day 11 or 18 and were provided with water for oviposition four days after feeding. Every treatment was replicated two or three times in each experiment (Figure 1). A replicate is a set of all treatments in which females of a single cohort (same age) were housed in one cage/treatment during blood feeding. Oviposition assays were performed on 8–10 females per (replicate) cage in each time point (on day 8, 15, and 22, Figure 1), thus providing a sample size of 16–30 per experiment. Hatch rate was measured for each egg batch that was laid.

Measures of egg batch size (EBS) and hatch rate were determined (see below). Females that died before their phenotype could be determined were excluded from analysis. The tubes were checked twice a day for three days; if eggs were laid, the female was killed and preserved and the appearance and number of eggs was recorded. Eggs that had sunk into the water were recorded separately and excluded from the count because they appeared abnormal and did not hatch in previous studies [20,21]. Eggs were then placed in labeled petri dishes and submerged in water. Hatch rate was recorded by transferring newly-hatched larvae onto filter paper via a glass pipette and counting them. Egg dishes were checked twice a day for three days to complete the estimate of hatch rate. Females that did not lay eggs or died during the experiment were dissected to determine their insemination status. Non-inseminated females were excluded from the analysis. Mosquito body size was estimated using the measurement of wing length as previously described [9]. Only females with at least one intact wing were measured.

The second experiment was carried out as stated above, with the addition of two treatment groups (each replicated twice). One treatment consisted of oviposition-deprived females that were provided with a second (supplemental) blood meal 4 days prior to receiving water for oviposition. The other treatment consisted of oviposition-deprived females, which received 100 4-day-old virgin males as supplemental mating. The males were kept in the cage for 3 days until the females were separated into oviposition tubes. A random collection of males from the same cohort and cages was placed with virgin females to assess their mating vigor. Virtually all virgin females were inseminated after 3 days (95%, n = 20). Individual females were separated and EBS and hatch rate were determined at the same time points as stated above.

### Ethics statement

This study was carried out in accordance with the recommendations in the Guide for the Care and Use of Laboratory Animals of the National Institutes of Health. All animal procedures were approved by the National Institutes of Health Animal Care and Use Committee (ACUC, Protocol ID: LMVR102).

### Data analysis

Statistical analyses were performed using SAS 9.2 (SAS Institute, Cary, NC). The effects of the treatments on EBS and hatch rate were evaluated using mixed model ANOVA (implemented using PROC MIXED) because both fixed-effect variables (e.g., oviposition-deprived groups vs. non-deprived control) and random variables (e.g., experiment and replicate nested within experiment) were included. If significant differences were indicated between treatments overall, contrasts were used to specifically evaluate the effect of oviposition-site deprivation compared with a subset of controls. Thus, to assess the effect of 7-day oviposition-site deprivation on EBS, contrasts were made between that group and the non oviposition-deprived controls which were in their 1^st^ and 2^nd^ gonotrophic cycle (1 contrast), and between the oviposition-deprivation group and the age-matched blood-deprivation group which received their first and only blood meal 3 days prior to the water for oviposition (1 contrast). The third contrast compared the EBS of the 1^st^ and 2^nd^ gonotrophic cycle of the non oviposition-deprived controls. Because all females laid no (zero) eggs in some treatment/replicate combinations, resulting in non-estimable hatch rates, the variance component associated with ‘replicate’ could not be estimated in such cases. To determine differences in the percentage of females that laid eggs from different groups, log-linear categorical models were implemented using PROC CATMOD, which allows testing the same contrasts.

## Results and discussion

The effect of oviposition-site deprivation on the reproductive output of *An. gambiae* was determined by comparison of EBS and egg-hatching rate of females subjected to short (7 days) and long (14 days) oviposition-site deprivation with corresponding values of non-deprived females of the same gonotrophic cycle as well as those of the same (chronological) age but in the next gonotrophic cycle. Additionally, oviposition-deprived females were compared to females of the same chronological age and same gonotrophic cycle, which received their first blood meal 3 days before they were offered water for oviposition; the last group of females was deprived of a blood meal (blood deprivation). Unless otherwise stated, data obtained in the two experiments were analyzed together, treating experiments as random blocks (see Methods).

### EBS

**Figure 2:**
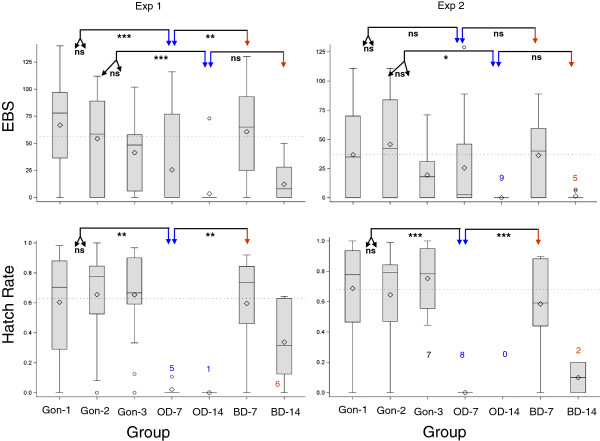
**Variation in egg batch size (EBS) and hatch rate within and between treatment groups across the two experiments.** Short-term (OD-7) and long-term (OD-14) oviposition-site deprived (OD) female groups are marked by the blue arrows and blood-deprived (BD) female groups are marked by red arrows. The dotted horizontal line denotes average across the first three gonotrophic cycles without deprivation. Sample sizes smaller than 10 are denoted by numbers with corresponding box-whisker plots. Analyses as described in Table 1 (EBS – 0 included and hatch rate), conducted on each experiment separately were used to compute the contrasts depicted by the arrows. In a box-whisker plot, lines and diamonds denote medians and means, respectively, ‘boxes’ mark quartiles, and ‘whiskers’ extend up to 1.5 times the inter-quartile range, while circles denote values beyond the whisker boundaries. Significance levels of ANOVA tests are denoted as: ns P>0.05; *P<0.05; **P<0.01; ***P<0.001.

In both experiments, long-term oviposition-site deprivation (14 days) reduced mean EBS several fold, to near zero, compared with that of the first and second gonotrophic cycles (Exp. 1: 3.5 vs. 67 and 54 eggs/female; Exp. 2: 0 vs. 45 and 37 eggs/female, respectively; Figure 2 and Table 1). However, mean EBS of 14-day oviposition-deprived females was not significantly smaller from that of 14-day blood-deprived females (12 and 1.4 eggs/female in experiments 1 and 2, respectively; Figure 2 and Table 1), suggesting that a nutritional deficit may mediate the effect of oviposition-site deprivation. Notably, mosquitoes in Exp. 2 were smaller (Additional file 1: Figure S1, P < 0.001, F_1,147_ = 150.8), which probably explains their smaller overall EBS (across groups) and reduced the statistical power due to smaller differences between groups. Even short-term oviposition-site deprivation (7 day) reduced mean EBS approximately 2-fold compared with that of the first and second gonotrophic cycles (Exp. 1: 26 vs. 67 and 54 eggs/female; Exp. 2: 26 compared with 45 and 37 eggs/female, respectively; Figure 2 and Table 1). Mean EBS of 7-day oviposition-deprived females was significantly smaller than that of 7-day blood-deprived mosquitoes in the first experiment (61 eggs/female; Figure 2), whereas minimal difference was detected between these groups in the second experiment.

**Table 1:** The effect of short-term (7 days) and long-term (14 days) oviposition-site deprivation on egg batch size (EBS) and hatch rate

		**EBS**			**EBS**	**0-exl**			**OvipRt **^**b**^		**HR**	
***Source***	***DF***	***Z/F***	***P***	***DF***	***Z/F***	***P***	***DF***	***χ***^***2***^	***P***	***DF***	***F***	***P***
Treatment group	6/238	12.1	0.001	6/138	6.72	0.001	6	34.5	0.001	4/127	12.9	0.001
Experiment	--	0.56	0.29		0.67	0.25	1	2.5	0.12	1/127	0.31	0.53
Replicate(Experiment)	--	0.77	0.22	--	ND	ND	--	ND	ND	--	ND	ND
Residual	--	10.9	0.001		8.31	0.001	6	6.8	0.34	127	--	--
(AIC) / (−2LL)^a^	--	(2433)	(2437)		1354	1350						
***Contrasts***												
OD-7 vs. Gon1 & Gon2	1/238	12.9	**0.001**	1/138	0.01	0.91		11.9	**0.001**	1/127	48.7	**0.001**
Gon1 & Gon2	1/238	0.23	0.63	1/138	0.20	0.65		0.0	0.97	1/127	0.03	0.86
OD-7 vs. BD-7	1/238	7.0	**0.009**	1/138	0.60	0.44		9.8	**0.002**	1/127	31.7	**0.001**
OD-14 vs. Gon1 & Gon3	1/238	31.9	**0.001**	1/138	0.04	0.83		16.8	**0.001**	--	ND	ND
Gon1 & Gon3	1/238	7.64	**0.006**	1/138	15.9	**0.001**		0.0	0.95	--	ND	ND
OD-14 vs. BD-14	1/238	0.6	0.44	1/138	2.64	0.11		7.1	**0.008**	--	ND	ND
OD-7 vs. OD-14	1/238	9.25	**0.003**	1/138	0.04	0.84		7.0	**0.008**	--	ND	ND

Mixed model ANOVA (Proc Mixed, SAS) was used to estimate variance among Experiments and Replicates (nested in Experiment) as well as Treatment group (fixed) on EBS. A log-linear categorical model (Proc Catmod, SAS) was used for oviposition rate. The effect of short-term oviposition-site deprivation on hatch rate was estimated using ANOVA (Proc GLM, SAS) using Experiment as random blocks. Hatch rate could not be estimated in females with long-term oviposition-site deprivation and blood deprivation because of very low oviposition rate (See also Figure 2 and the Methods for more details). Bold values were significant after accommodating the number of contrast tests (for each response variable separately) using the Sequential Bonferroni procedure [22].

^a^ Model fit statistics residual −2 Log Likelihood and AIC values are given in parentheses in the DF column.

^b^ Log-linear categorical models (Proc Catmod, SAS) were used to assess the effects of various factors.

The proportion of oviposition-site deprived females that laid eggs (oviposition rate) was significantly reduced compared with non-deprived females, but the difference in mean EBS between the oviposition-site deprived and non-deprived females in those that laid eggs was minimal (Table 1 and Additional file 2: Figure S2). These results suggest that the overall effect of oviposition-site deprivation on EBS (including zero eggs) was mostly due to a lower oviposition rate rather than to a reduced number of eggs in those females that laid eggs. A high proportion of the oviposition-deprived females that did not lay eggs, especially those subjected to long oviposition deprivation, retained deformed brownish eggs that appeared to be undergoing resorption.

### Hatch rate

**Figure 3:**
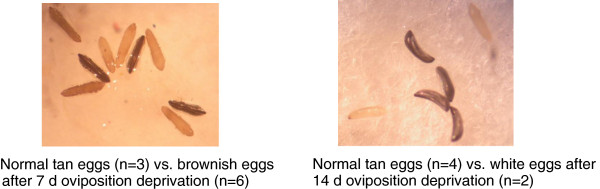
**Eggs of females that experienced short (Left) and long (Right) oviposition-site deprivation with normal eggs of non-deprived females for comparison.** Note that eggs fail to tan as the length of deprivation increases. Tests significance levels are denoted as in Figure 2.

In both experiments, nearly all females subjected to long (14 day) oviposition-site deprivation laid no eggs (see above), precluding estimating hatch rate for these groups. Thus, hatch rate was analyzed considering short-term (7-day) oviposition-site deprivation only. Remarkably, short-term oviposition-site deprivation reduced hatch rate to 0-2% from 60-75% in both experiments (Figure 2 and Table 1). Associated with the reduced hatch rate was a high frequency of brownish eggs whose shell was sunk (Figure 3), similar to previous observations, e.g., [16]. However, many normal-looking eggs also failed to hatch.

### The roles of nutrition and sperm viability on egg batch size and hatch rate

**Figure 4:**
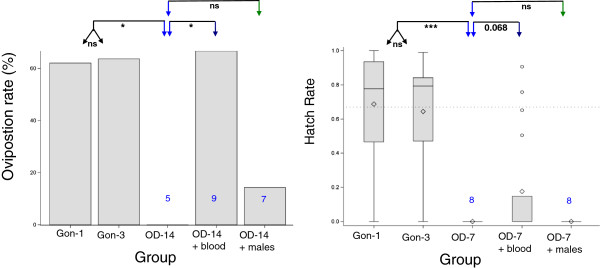
**The effects of a supplemental blood meal (purple) and supplemental insemination (green) on the egg-laying response (oviposition rate) of oviposition-site deprived (OD) females (blue).** One-sided tests were used to evaluate if the supplemental treatments increased oviposition rate, but a two-sided test was used to compare the two supplemental treatments to each other. Tests are marked by lines with arrows. Sample sizes smaller than 10 are denoted by numbers with corresponding box-whisker plots.

The underlying role of nutrition and sperm viability in mediating the effect of oviposition-site deprivation on EBS and hatch rate were evaluated in experiment 2 by providing oviposition-site deprived females with a supplemental blood meal or supplemental “insemination” via access to males, three days prior to provision of water for oviposition. The males used in the “supplemental insemination” treatment were randomly selected from others that mated successfully with virgin females. The oviposition rate of females subjected to long-term oviposition-site deprivation “recovered” to values typical of non-deprived females after they received one supplemental blood meal (the oviposition rate of supplemented females was slightly higher than that of non-deprived females), but not after they received “supplemental insemination” (Figure 4). The hatch rate after short term oviposition-site deprivation apparently increased from zero to 20%, but this increase was only marginally significant (P < 0.069, Figure 4). However, as with egg batch size, no increase in hatch rate was indicated following the supplemental “insemination” treatment (Figure 4).

## Conclusions

The sensitivity of *An. gambiae* to short-term oviposition-site deprivation suggests that even short dry spells (measured in days) during the wet season can reduce reproductive success, especially because of lower fertility (hatch rate) augmented by lower fecundity (egg batch size) due to a lower rate of oviposition. Similar sensitivity was reported for other culicids including *An. pharoensis* and *An. maculatus*[7,15-18]. High sensitivity to only a few days of oviposition-site deprivation is surprising for species that are often subjected to short (and long) dry spells under natural conditions, as is the case for *An. gambiae*. It is possible that a colony adapted to laboratory conditions has increased its sensitivity to oviposition-site deprivation because such deprivation is too rare in an insectary and it may also be related to other factors such as different blood source (chicken instead of human in our colony). However, the agreement across anopheline species (above), especially with the arid-adapted *An. pharoensis,* which was raised in the laboratory only three years prior to the study and was provided with human blood, suggests that the sensitivity to oviposition-site deprivation reflects the natural response of anophelines to such pressure.

However, oviposition-site deprivation was linked, as in most other studies listed above, with no (or limited) access to blood meals. The capacity to regain reproductive success by supplemental blood feeding suggests that, under natural conditions, mosquitoes may avoid the effects of oviposition-site deprivation by increasing their blood-feeding frequency. This hypothesis is consistent with the observations of gravid females that take additional blood meals [14,23,24]. Thus, malaria transmission may not decline (and might even increase) during a short dry spell. Additional studies measuring the blood-feeding rate of oviposition-site deprived *An. gambiae* females are required to clarify that hypothesis.

The importance of oviposition-site deprivation as a cue to alter the physiology of *An. gambiae* during the long dry season is not evident from these results. Similar to this study, Yaro *et al.* (2012) [14] reported a reduced oviposition rate in field-collected (blood-fed, semi-gravid, or gravid) females that were provided with water for oviposition once they reached the gravid state (without experimental deprivation) in dry-season females as compared with their wet-season counterparts. Presumably these dry-season females blood fed while they were naturally deprived of oviposition sites. Unlike the present study, the egg batch size for females that laid eggs was also lower in the Sahel [14] and a high frequency of brownish eggs was not observed [14]. Although their study did not empirically measure hatch rate, the dramatic reduction in hatch rate was not observed during the dry season (Yaro, Alpha and Dao, Adama - unpublished data). These observations suggest that during the long dry season in the Sahel, altered female physiology modifies the effect of oviposition-site deprivation on their reproductive output. This hypothesis requires additional study, as well as the effects of oviposition site deprivation on survival and on the vectorial competence (susceptibility) with human pathogens, as was recently studied in *Culex pipiens quinquefasciatus* and West Nile Virus [25].

## Competing interests

The authors declare that they have no competing interests.

## Authors’ contributions

KLD participated in the study design, carried out the experiments, and helped draft the manuscript. DLH participated in the study design and helped carry out various aspects of the experiments as well as draft the manuscript. TL conceived of the study, participated in its design and coordination, conducted the statistical analysis, and drafted the manuscript. All authors read and approved the final manuscript.

## Supplementary Material

Additional file 1 **Figure S1.** Variation in body size, measured by wing length (WL), within and among treatment groups in the two experiments. The dotted horizontal line denotes the average WL across all groups of each experiment. Sample sizes smaller than 10 are denoted by numbers under corresponding box-whisker plots. Click here for file

Additional file 2 **Figure S2.** Partitioning the effect of oviposition-site deprivation on overall egg batch size (EBS; upper panel), the number of eggs per batch for females that laid eggs (middle panel), and oviposition rate (lower panel). EBS of the treatment groups was averaged across experiments (see Figure 2 for results by experiment), following insignificant variance between experiments (see text and Table 1). Oviposition rate is shown separately for each experiment. Means of treatment groups with different letters are statistically different. Click here for file
