# Supplementary figures and images for: The effects of oviposition-site deprivation on Anopheles gambiae reproduction
Source: Parasit Vectors. 2012 Oct 16;5:235. doi: 10.1186/1756-3305-5-235 (PMC3514158; doi:10.1186/1756-3305-5-235)

## Slide 1
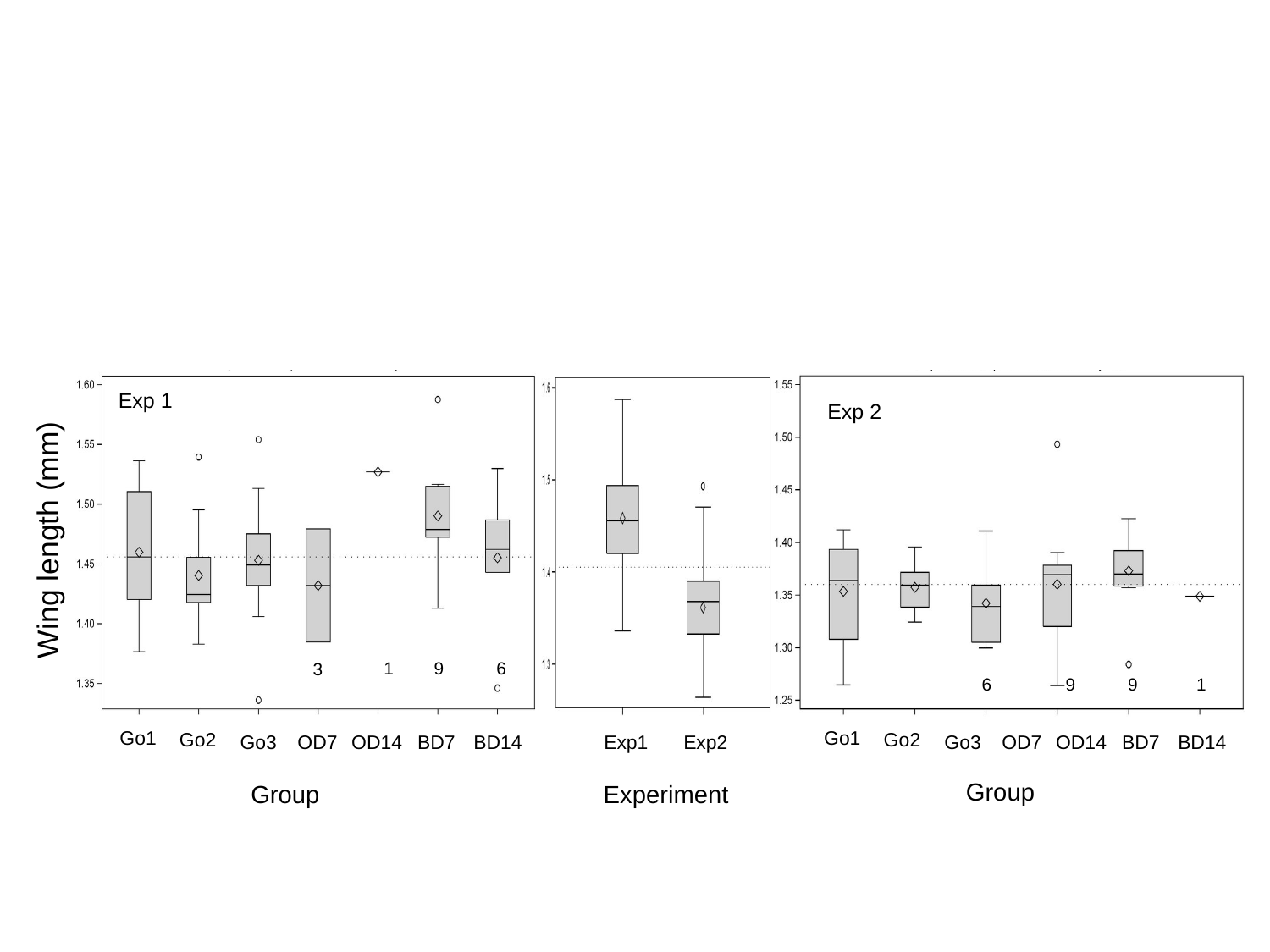

Exp 1
Exp 2
Wing length (mm)
1
9
6
3
9
9
1
6
Go1
Go1
Go2
Go2
Go3
OD7
OD14
BD7
BD14
Exp2
Go3
OD7
OD14
BD7
BD14
Exp1
Group
Group
Experiment

Supplement: Additional file 1 — Figure S1. Variation in body size, measured by wing length (WL), within and among treatment groups in the two experiments. The dotted horizontal line denotes the average WL across all groups of each experiment. Sample sizes smaller than 10 are denoted by numbers under corresponding box-whisker plots. [file 1756-3305-5-235-S1.ppt]
