# Supplementary material for: The effects of oviposition-site deprivation on Anopheles gambiae reproduction
Source: Parasit Vectors. 2012 Oct 16;5:235. doi: 10.1186/1756-3305-5-235 (PMC3514158; doi:10.1186/1756-3305-5-235)
Supplement: Additional file 2 — Figure S2. Partitioning the effect of oviposition-site deprivation on overall egg batch size (EBS; upper panel), the number of eggs per batch for females that laid eggs (middle panel), and oviposition rate (lower panel). EBS of the treatment groups was averaged across experiments (see Figure 2 for results by experiment), following insignificant variance between experiments (see text and Table 1). Oviposition rate is shown separately for each experiment. Means of treatment groups with different letters are statistically different. [file 1756-3305-5-235-S2.ppt]

## Slide 1
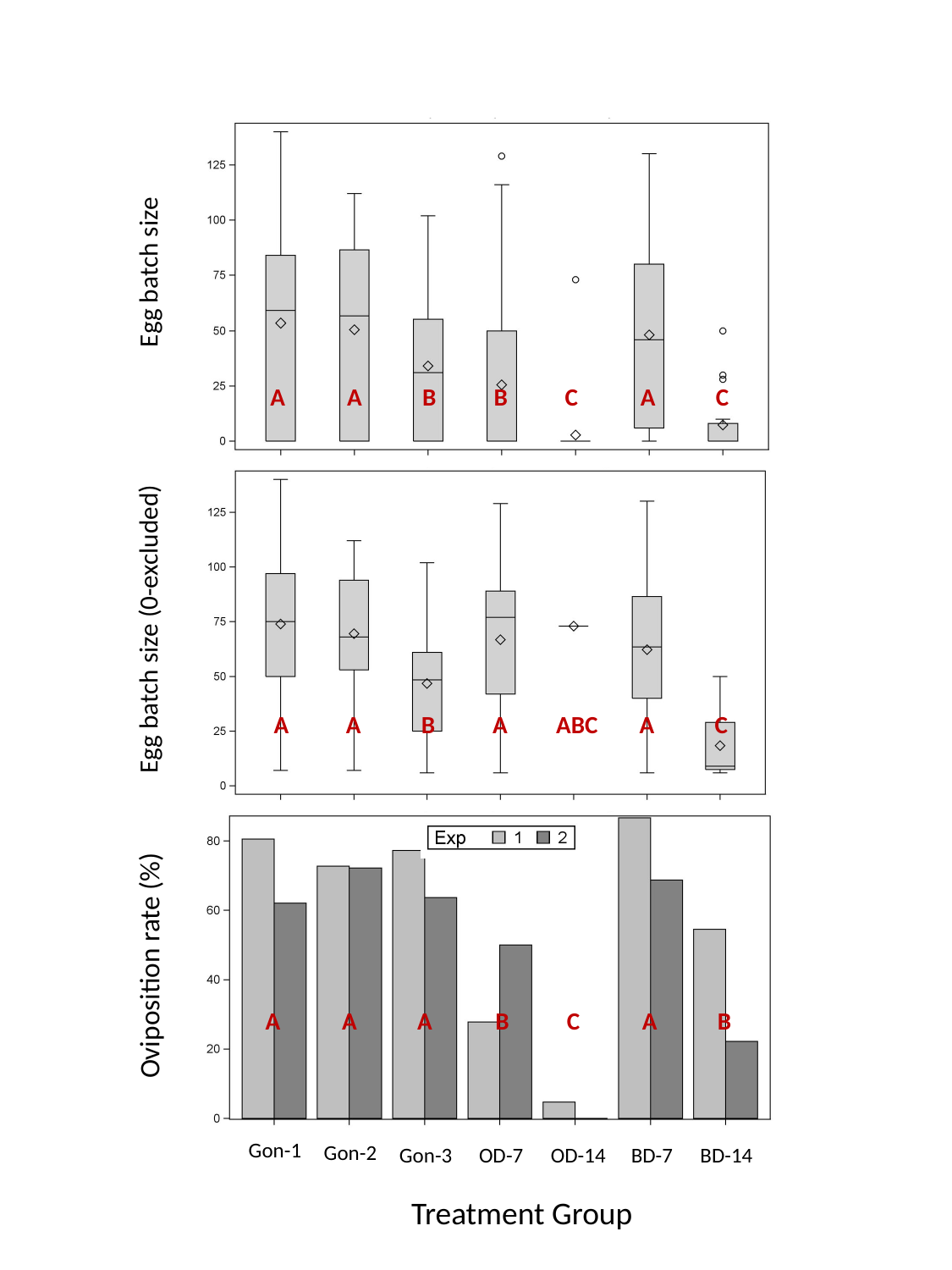

Egg batch size
A
A
B
B
C
A
C
Egg batch size (0-excluded)
A
A
B
A
ABC
A
C
Oviposition rate (%)
A
A
A
B
C
A
B
Gon-1
Gon-2
Gon-3
OD-7
OD-14
BD-7
BD-14
Treatment Group
